# Testing the interaction between analytical modules: an example with Roundup Ready^® ^soybean line GTS 40-3-2

**DOI:** 10.1186/1472-6750-10-55

**Published:** 2010-08-05

**Authors:** Gianni Bellocchi, Marzia De Giacomo, Nicoletta Foti, Marco Mazzara, Eleonora Palmaccio, Cristian Savini, Chiara Di Domenicantonio, Roberta Onori, Guy Van den Eede

**Affiliations:** 1 European Commission, Joint Research Centre, Institute for Health and Consumer Protection, Molecular Biology and Genomics Unit, via E. Fermi 2749, 21027 Ispra (VA), Italy; 2 Italian National Institute of Health, Department of Veterinary Public Health and Food Safety, GMO and Mycotoxins Unit, viale Regina Elena 299, 00161 Rome, Italy

## Abstract

**Background:**

The modular approach to analysis of genetically modified organisms (GMOs) relies on the independence of the modules combined (i.e. DNA extraction and GM quantification). The validity of this assumption has to be proved on the basis of specific performance criteria.

**Results:**

An experiment was conducted using, as a reference, the validated quantitative real-time polymerase chain reaction (PCR) module for detection of glyphosate-tolerant Roundup Ready^® ^GM soybean (RRS). Different DNA extraction modules (CTAB, Wizard and Dellaporta), were used to extract DNA from different food/feed matrices (feed, biscuit and certified reference material [CRM 1%]) containing the target of the real-time PCR module used for validation. Purity and structural integrity (absence of inhibition) were used as basic criteria that a DNA extraction module must satisfy in order to provide suitable template DNA for quantitative real-time (RT) PCR-based GMO analysis. When performance criteria were applied (removal of non-compliant DNA extracts), the independence of GMO quantification from the extraction method and matrix was statistically proved, except in the case of Wizard applied to biscuit. A fuzzy logic-based procedure also confirmed the relatively poor performance of the Wizard/biscuit combination.

**Conclusions:**

For RRS, this study recognises that modularity can be generally accepted, with the limitation of avoiding combining highly processed material (i.e. biscuit) with a magnetic-beads system (i.e. Wizard).

## Background

To comply with the European regulation concerning the labelling and traceability of genetically modified organisms (GMOs) and GM products [1] and offer freedom of choice to consumers, development of reliable, sensitive and accurate methods for GMO detection and quantification in food, feed and raw materials is essential [2]. Measurement of deoxyribonucleic acid (DNA) by polymerase chain reaction (PCR) has been widely used in different fields of food analysis and real-time PCR is the established preferred method for quantification of GMOs [3-5]. This is reflected in the EU legislation establishing the European Union Reference Laboratory [1], hereinafter referred to as the EURL for Genetically Modified Food and Feed (EURL-GMFF), whose main task is to validate methods for detection of GMOs in order to ensure full traceability along the food and feed chain [6] and that methods cover all the steps needed, including DNA extraction and subsequent quantification by PCR [7]. The whole analytical procedure for GMO quantification in food and feed consists of several, add-on sequential steps, from sample preparation to DNA extraction, purification and real-time PCR measurement. The result is provided in the form of a ratio between the GM- and the species-specific target sequences, preferably expressed in terms of haploid genomes [8,9].

The modular approach looks at an analytical method as a combination of different procedural steps in the analytical chain (the 'modules') and is suitable to rationalise both validation and application of methods for GMO detection [10]. Modularity implies independence of modules, therefore it allows for flexibility to combine modules on the one hand and for harmonisation on the other hand. If modular validation is to be applied, fit-for-purpose procedures and general acceptance of minimum requirements for each module are needed in order to evaluate the uncertainties associated with each module.

Measurement of the uncertainty is relevant to assess the quality of the analytical result. The uncertainty components of various modules are currently being evaluated at different levels by several groups (the European Network of GMO Laboratories, the Consultative Committee for Amount of Substances Metrology in Chemistry, Codex Alimentarius, the European Committee for Standardisation [CEN], and the International Organisation for Standardisation [ISO]). Nevertheless, despite these efforts on international standardisation, the minimum quality requirements that extracted DNA should meet in order to be fit for the analytical module consisting of quantitative real-time PCR measurement (qPCR) should be clearly defined and experimentally corroborated.

Previous investigations suggested that different extraction methods could influence DNA quantification in food products by qPCR [11,12]. Because PCR requires a high-quality DNA template in terms of DNA integrity and purity, the method used to extract the DNA from the starting material is critical, as pointed out by other Authors [13,14]. Other studies have demonstrated that quantification of GMOs was affected by the degree of processing of the matrix from which genomic DNA (gDNA) was extracted [15-17]. Similar influences on GMO quantification have been reported for processed corn [18].

The modular approach was proposed to facilitate validation of the procedure for GMO measurement [10]. This pragmatic approach implies that each step (module) in the analytical procedure, i.e. DNA extraction and determination of the DNA concentration, can be decoupled either from the previous or from the next one, provided that each step fulfils certain quality criteria. The advantage of such an approach lies in the fact that separately validated modules, for example for DNA extraction or for real-time PCR, could then be combined. The same approach has also been taken in ISO standards on GMO analysis [19,20]. Moreover, if the real-time PCR measurement is not influenced by the type of extraction method applied it can be validated with DNA extracted by any method and from any type of matrix, although scientific evidence for this has yet to be produced.

In this pilot study, the EURL-GMFF's validated method for detection of the world's leading GM soybean crop [21] - glyphosate-tolerant Roundup Ready soybean (unique identifier MON-Ø4Ø32-6) - was chosen as a model. The study is intended as a proof of concept for testing whether the DNA extraction methods applied to a given matrix prior to qPCR analysis can affect quantification of GMO in food or feed and to investigate which, if any, criteria should be applied in order to make the qPCR module independent from the DNA extraction module.

## Methods

### Sample material

Powdered certified reference material (CRM) for genetically modified soybean line 40-3-2 (1% RRS ERM-BF410d) was provided by the European Commission's Institute for Reference Materials and Measurements (IRMM, Geel, Belgium) and is commercialised by Fluka http://www.sigmaaldrich.com/analytical-chromatography/fluka-analytical/about-fluka-and-riedel.html. Feed meal (ingredients: soybean flour, wheat bran, dried beet pulp, beans, sugar beet molasses, calcium carbonate, magnesium oxide, sodium hydrogen carbonate and palm oil) was purchased in a local store.

Biscuit samples, kindly provided by the University of Parma (UPAR), Italy were prepared as follows: 100% RRS flour was mixed with non-GM RRS flour (provided by Progeo Molini S.p.A, Italy: http://www.progeomolini.it) to obtain a 1% GM mixture. Subsequently, the mixture was used for biscuit production with the following ingredients: 200 g of wheat flour, 100 g of mixed RRS flour, 50 g of sugar, 7 g of yeast and 200 g of water, stirred just until the ingredients were moistened and the dough formed a soft ball. The dough was kneaded until smooth. Biscuits were cut out, each weighing 15 grams. The biscuits were baked in an oven at 180 °C for 10 minutes.

### Data generation

**Figure 1:**
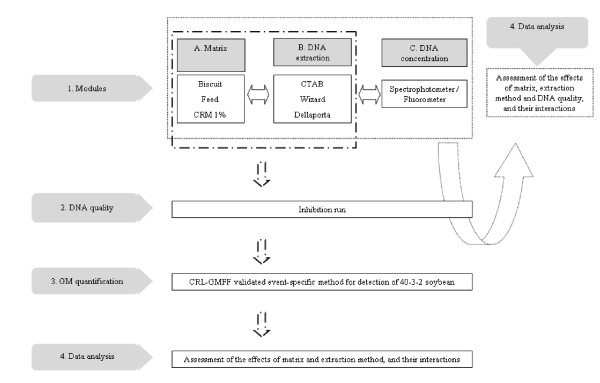
**Experiment design followed to test for module independence**.

An experimental plan was designed by the ISS (Rome, Italy) and the EURL-GMFF (Joint Research Centre, European Commission, Ispra, Italy) to evaluate the effect of alternative DNA extraction methods applied to matrices (and possible interaction effects) on determination of the content of Roundup Ready soybean (RRS, line GTS-40-3-2) relative to total soybean DNA (see Figure 1).

Three matrices containing Roundup Ready soybean were selected. Feed and biscuit contained the GM ingredient at unknown DNA concentrations. The third matrix was certified reference material containing 10 g kg^-1 ^of Roundup Ready soybean (CRM 1%, ERM-BF410d). The samples tested were selected to reproduce a range of real cases from unprocessed grain/flour (CRM 1%) to feed meal generally present on the market in complex feed formulations and a heavily consumed bakery product for humans. Total DNA was extracted at ISS (six extractions from each matrix in the same day) from 200 mg of each of the above-mentioned matrices by three well-known and widely used extraction methods, based on different purification and extraction mechanisms:

- the CTAB-based protocol ('CTAB') a cellular lysis buffer, and selective precipitation with cetylammonium bromide [20];

- the Dellaporta-derived method ('Dellaporta') uses first a lyses step (thermal lyses in the presence of Tris HCl, EDTA, CTAB and β-mercaptoethanol) followed by deprotenisation and removal of contaminants by phenol-chloroform precipitation [22]. The method has been validated by EURL-GMFF in the context of method validation for specific quantification of genetically modified soybean line Roundup Ready^®^. The protocol requires extraction of 6 g of sample, but in the case of the CRM 1%, given its homogeneity and stability, the quantity was reduced to 200 mg, with all the reagents scaled down accordingly. No changes were made to the protocol to extract DNA from the other matrices http://gmo-crl.jrc.ec.europa.eu/summaries/A2704-12_soybean_DNAExtr_report.pdf;

- and the Promega (Promega Italia s.r.l., Milan, Italy) Wizard™ ('Wizard') magnetic DNA purification for food kit [23]. The method uses a lyses step (thermal lyses in presence of Tris HCl, EDTA and SDS) and applies the Promega's MagneSil magnetic bead technology for the DNA isolation based on silica as affinity matrix in a mobile solid phase.

The extraction methods were selected according to their wide application in GMO analyses, the difference on lyses buffer and on DNA isolation technique.

The concentration of the extracted DNA was determined at the EURL-GMFF, by fluorescence detection using PicoGreen dye for dsDNA quantification with a BioRad VersaFluor fluorometer (BioRad, Milan, Italy), and at the ISS, spectrophotometrically by NanoDropTM ND-1000 (Celbio s.p.a., Milan, Italy). Spectrophotometric measurements were performed at wavelengths of 230, 260 and 280 nm. Absorbance ratios at 260:280 nm and 260:230 nm were used to assess the detection of protein impurities or other contaminants that absorb strongly at or near 280 nm (with a 260:280 ratio < 1.8 and > 2.0) and impurities of phenol or other organic compounds (with a 260:230 ratio < 2.0) co-extracted with the nucleic acids from the specimens [24].

### Acceptance criteria for DNA quality

DNA extracts were analysed by electrophoresis of 1% agarose gel pre-stained with ethidium bromide. Two microlitres of each extract were mixed with tracer dye - 6X DNA loading dye (Fermentas Cat. #R0611) - and an appropriate amount of distilled water, loaded in the gel and run at 70V. Lambda DNA digested with EcoRI and Hind III was used as the molecular size marker (Promega Cat. #G1731). A gel camera (Bio-Rad Gel Doc 2000) provided the digital photograph recording system.

Three performance criteria need to be met before DNA extracts can be accepted: DNA concentration needs to meet the requirement of the validated method; the DNA is of sufficient length to be amplified by PCR; and it is adequately free from PCR inhibitors. In the case of the latter, the DNA quality was demonstrated by analysing in duplicate four four-fold serial dilutions of each DNA extract (inhibition runs) using the validated soybean-specific reference system (lectin gene: http://gmo-crl.jrc.ec.europa.eu/summaries/40-3-2_validated_Method.pdf). First, a four-fold dilution series is prepared (from 1:4 to 1:256). To assess the presence of inhibitors, the Ct values of the diluted samples are plotted against the logarithm of the dilution factor and an equation is calculated by linear regression. Moreover, the Ct value of the 'undiluted' sample extrapolated from the linear regression is compared with the Ct measured from the same sample. The conditions to be met before DNA extracts can be accepted are: the slope of the regression line must be between -3.6 and -3.1; the linearity (*R^2^*) must be equal to or above 0.98; and the difference between the measured Ct and the extrapolated Ct value (ΔCt) must be within 0.5. This approach has been endorsed by the European Network of GMO Laboratories (ENGL: http://engl.jrc.ec.europa.eu) and validated by the EURL-GMFF [25].

The content of Roundup Ready^® ^soybean relative to the total DNA content in the DNA extracts was quantified using a validated Roundup Ready^® ^soybean line-specific method http://gmo-crl.jrc.ec.europa.eu/summaries/40-3-2_validated_Method.pdf. To determine the % RRS soybean content in samples, four microlitres of DNA extracts were loaded per reaction, up to a maximum amount of 200 ng of DNA based on estimates from fluorimetric means). Each DNA extract was analysed in triplicate and the relative GM content was calculated by generating two standard curves (one for the reference sample, the other for the RRS system) plotting the Ct values measured for the calibration points. Calibration curves were built by DNA copy number, starting from a 10% GM content in the first sample and then serially diluting to encompass more than two orders of magnitude.

To determine the amount RRS DNA in the DNA extract, the RRS copy number was divided by the copy number of the soybean reference gene (lec) and multiplied by 100 to obtain the percentage value (GM% = RRS/lec * 100).

### Experiment design and data processing

Both the DNA yield and the % GM DNA quantification were processed using SAS procedures (version 9, SAS Inst. Inc., Cary, NC, http://www.sas.com). PROC UNIVARIATE provided an array of tests (Shapiro-Wilk, Kolmogorov-Smirnov, Cramer-von Mises and Anderson-Darling) for departure of data distribution from normal plus graphic tools (box plot and stem-and-leaf representation) to detect outlying data. Then analyses of variance (PROC GLM) were performed by SAS to construct tests to determine the significance of classification effects. One typical goal was to compare the means of the response variables (DNA yield and % GM DNA) for various combinations of the classification variables. In particular, two-way analysis of variance was applied to investigate the effect on the % RRS values of the extraction method, the matrix and the interaction between them. For DNA measurements, a three-way analysis was performed to include the effect of the method for determining the DNA concentration (fluorometric or spectrophotometric). The basic factors were arranged in a fully crossed experiment design (see Table 1). The Levene test was used to assess departures from homogeneous variances. One-factor conventional and Welch's analyses of variance were also run. Welch's non-parametric approach is robust to departures from non-homogeneity of variance and is assumed to be more reliable than conventional analysis of variance under such conditions. The Ryan-Einot-Gabriel-Welch multiple range test (REGWQ) was used for post-hoc comparisons for overlapping groups of means.

**Table 1:** DNA measurements and GMO values (% RRS) for all DNA extraction method/matrix combinations.

Extraction method	Matrix	260:280 nm ratio	260:230 nm ratio	Spectrophotometric DNA yield (μg g^-1^)	Inhibition run	% RRS	Fluorescence DNA yield (μg g^-1^)
CTAB	Feed	1.89	2.42	114.0	Ok	0.90	84.5
	Feed	1.87	2.39	133.5	Ok	0.88	101.0
	Feed	1.88	2.34	127.0	Ok	0.78	99.0
	Feed	1.86	2.42	121.0	Ok	0.63	94.0
	Feed	1.87	2.35	144.0	Out of AC*	0.82	114.0
	Feed	1.89	2.42	103.5	Ok	1.11	80.0
	
	Biscuit	1.6	1.64	28.5	Ok	1.74	7.5
	Biscuit	1.38	1.32	12.5	Ok	1.55	3.0
	Biscuit	1.41	1.17	10.0	Out of AC	2.04	3.0
	Biscuit	1.63	1.63	25.5	Ok	1.19	6.5
	Biscuit	1.58	1.71	24.5	Ok	1.40	7.5
	Biscuit	1.3	1.10	10.0	Ok	1.32	2.5
	
	CRM 1%	1.67	1.87	69.5	Ok	1.31	56.0
	CRM 1%	1.70	1.90	45.5	Ok	1.34	38.5
	CRM 1%	1.73	2.15	65.0	Ok	1.01	59.0
	CRM 1%	1.69	1.92	43.0	Ok	1.14	36.0
	CRM 1%	1.69	1.95	41.0	Ok	1.45	47.5
	CRM 1%	1.73	2.05	67.0	Ok	1.09	81.0

Wizard	Feed	1.70	0.87	83.0	Out of AC	1.31	32.5
	Feed	1.77	0.67	105.5	Out of AC	0.96	38.0
	Feed	1.66	0.92	118.0	Out of AC	1.50	33.5
	Feed	1.86	0.27	70.0	Ok	1.07	37.5
	Feed	1.79	1.05	49.0	Out of AC	1.35	24.8
	Feed	1.72	1.10	68.0	Out of AC	1.35	28.3
	
	Biscuit	1.77	0.39	92.0	Ok	3.29	4.1
	Biscuit	1.84	0.13	11.5	Ok	3.06	1.7
	Biscuit	1.86	0.18	30.5	Ok	4.90	2.8
	Biscuit	1.83	0.34	64.0	Out of AC	3.15	2.6
	Biscuit	1.8	0.31	84.0	Ok	2.03	3.2
	Biscuit	1.86	0.17	62.5	Ok	2.11	3.4
	
	CRM 1%	1.59	0.23	62.0	Out of AC	1.04	3.7
	CRM 1%	2.02	0.21	46.5	Ok	1.45	10.9
	CRM 1%	1.98	0.17	56.5	Ok	0.58	4.1
	CRM 1%	2.09	0.04	14.5	Ok	1.08	5.4
	CRM 1%	1.87	0.27	86.5	Out of AC	1.34	9.6
	CRM 1%	1.91	0.08	29.0	Ok	1.31	12.2

Dellaporta	Feed	2.19	0.18	72.0	Out of AC	0.99	10.3
	Feed	2.05	0.27	111.0	Ok	0.90	24.8
	Feed	2.07	0.27	107.5	Ok	0.94	22.8
	Feed	1.94	0.61	294.0	Out of AC	0.80	61.0
	Feed	2.17	0.2	78.5	Ok	0.86	14.8
	Feed	2.75	0.09	34.5	Ok	0.71	7.8
	
	Biscuit	2.27	0.11	62.5	Ok	1.15	4.5
	Biscuit	2.87	0.05	29.0	Ok	1.13	4.2
	Biscuit	2.39	0.08	43.5	Ok	1.26	3.0
	Biscuit	2.83	0.05	29.5	Ok	1.40	4.0
	Biscuit	2.58	0.06	32.0	Ok	0.96	3.0
	Biscuit	2.58	0.06	34.5	Ok	1.01	4.0
	
	CRM 1%	1.90	2.11	74.0	Ok	1.03	42.5
	CRM 1%	1.91	2.25	78.5	Ok	1.14	33.0
	CRM 1%	1.71	1.89	21.5	Out of AC	0.93	15.0
	CRM 1%	1.58	0.73	40.5	Out of AC	1.06	17.7
	CRM 1%	1.78	4.25	45.0	Ok	1.25	22.2
	CRM 1%	1.84	1.94	20.0	Out of AC	1.25	2.8

* 'Out of AC' stands for data outside the acceptance criteria for DNA quality from inhibition runs

For % GM DNA quantification, statistical results were generated from the analyses described above by either including or not including the data not complying with the DNA quality criteria recommended by the EURL-GMFF.

For feed and biscuit, the true % GM content was unknown and therefore estimated based on the log-normal distribution for the variation associated with PCR-based measurements [e.g. [26]]. For each matrix and DNA extraction method, two ISO validation metrics (average percent bias - *B *- and percent repeatability standard deviation - *RSD_r_*) were calculated and, together with PCR amplification efficiency rates (*E_1 _*- reference gene efficiency and *E_2 _*- target gene efficiency), combined into a modular, synthetic fuzzy-based indicator. In particular, two aggregation modules were defined - accuracy and efficiency - combining validation metrics and efficiency rates respectively. In both cases, the basic measures were given equal importance (expert weight equal to 0.50) in terms of affecting the relevant module. The two modules were then aggregated into an indicator giving greater importance to accuracy (0.75) than efficiency (0.25). This approach relies on the rationale and expert settings outlined by [27] and use of AMPE software for the calculations [28].

## Results

A programme of experiments was designed by the ISS (Rome, Italy) and EURL-GMFF (Joint Research Centre, European Commission, Ispra, Italy) to evaluate the effect of alternative DNA extraction methods applied to matrices on determination of the content of Roundup Ready^® ^soybean (RRS, line GTS-40-3-2) relative to total soybean DNA (see Figure 1).

**Figure 2:**
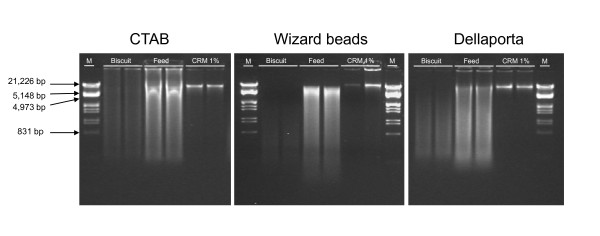
**Agarose-gel electrophoresis of DNA extracts by matrix and DNA extraction method**. M, λ DNA/EcoRI + HindIII.

DNA extracted from the three matrices (biscuit, feed and CRM 1%) according to three mentioned DNA extraction methods were first analysed by agarose-gel electrophoresis, where two microlitres of the samples were loaded and analysed by comparison with molecular size markers (see Figure 2). This way, a comparison of the nucleic acids extracted by the same extraction procedure from the different matrices, i.e. biscuit, feed and CRM 1%, is readily available. DNA from complex matrices such as biscuit and feed show signs of extensive degradation with all the procedures. Commonly used DNA isolation methods such as 'CTAB' and 'Della Porta' provide higher DNA yield when applied to feed material and, in decreasing order, to CRM 1% and biscuit.

Roundup Ready^® ^soybean amplicon has a length of 84-bp, whilst the soybean-specific reference amplicon - based on a lectin gene - has a length of only 74-bp http://gmo-crl.jrc.ec.europa.eu/summaries/40-3-2_validated_Method.pdf, thus in the present study average length of DNA fragments are well above the target amplicon size, though less visible for Wizard extracts from CRM 1% matrix; this ensures amplifiability of target analytes as demonstrated by amplification data from inhibition runs (outcomes shown) and quantification (% RRS) of the target analyte in qPCR (Table 1). Data on the DNA concentration (by spectrophotometer and PicoGreen) and GMO quantification (determination of % RRS) for three matrices (feed, biscuit and CRM 1%) for three DNA extraction methods (CTAB, Wizard and Dellaporta) are summarised in Table 1, which also shows the absorbance ratios (260:280 and 260:230) and outcomes from inhibition runs. DNA yields are given as weight/weight ratios between DNA and the matrix.

For several samples the absorbance ratios fall outside the criteria adopted as an indication of DNA purity (260:280 between 1.8 and 2.0; 260:230 ≥ 2.0). CTAB/feed was the only combination in which impurities from proteins were not found. Wizard showed far less than acceptable absorbance ratios in the nucleic acid preparations, thus pointing to consistent impurities with any matrix. With Dellaporta, impurities were observed, especially when applied to feed and biscuit matrices. In general, clues to impurity from absorbance ratios tend not to be related to the acceptance criteria for inhibition runs.

Inhibitory effects were revealed in more than 40% of the cases with Wizard, in about 30% with Dellaporta and in about 10% with CTAB. Similar percentages were observed with matrices, feed being the most inhibited and biscuit the least. The worst combination was Wizard extraction from a feed matrix (five out of six samples inhibited). No inhibitory effects were observed with either CTAB on CRM 1% or with Dellaporta on biscuit.

DNA yield (μg g^-1^), estimated from spectrophotometric determination of the DNA concentration, showed a moderate departure from normal (*P *< 0.05) only with the Dellaporta/feed combination, with some bias towards high values (positive asymmetry). Estimates of RRS concentration in DNA extracts (% RRS in Table 1) were normally distributed with all the tests (*P *> 0.05), but some violations of the assumption of equal variance were identified (Levene test, *P *< 0.01).

With both DNA yield and DNA concentration, comparisons with one-way conventional analysis of variance revealed significant differences (*P *< 0.01) between both factors. The same responses were confirmed by non-parametric Welch's analysis of variance. Hence, in spite of heterogeneous variances, conventional one-way ANOVA and the non-parametric approach proved equally reliable. For this reason, the conventional parametric approach was applied in the fully crossed three-way (DNA yield) and two-way (% GM DNA) analyses of variance (Tables 2 and 3, respectively).

**Table 2:** DNA yield - three-way analysis of variance

Source of variation	Degrees of freedom	F value	Probability
Total	107		
Extraction (of DNA)	2	5.7	0.0047
Matrix	2	44.2	< 0.0001
Concentration (of DNA)	1	54.3	< 0.0001
Extraction/Matrix	4	5.6	0.0005
Extraction/Concentration	2	5.1	0.0079
Matrix/Concentration	2	3.8	0.0257
Extraction/Matrix/Concentration	4	1.38	0.2484
Residual	90		

**Table 3:** % GMO (% RRS): two-way analysis-of-variance

Source of variation	All data			Excluding 'out of AC'* data		
	
	Degrees of freedom	F value	Probability	Degrees of freedom	F value	Probability
Total	53			38		
Extraction (of DNA)	2	19.49	< 0.0001	2	4.94	0.0140
Matrix	2	28.41	< 0.0001	2	13.45	< 0.0001
Extraction/Matrix	4	11.29	< 0.0001	4	6.69	0.0006
Residual	45			31		

* 'Out of AC' stands for data outside the acceptance criteria for DNA quality from inhibition runs

### DNA yield

**Figure 3:**
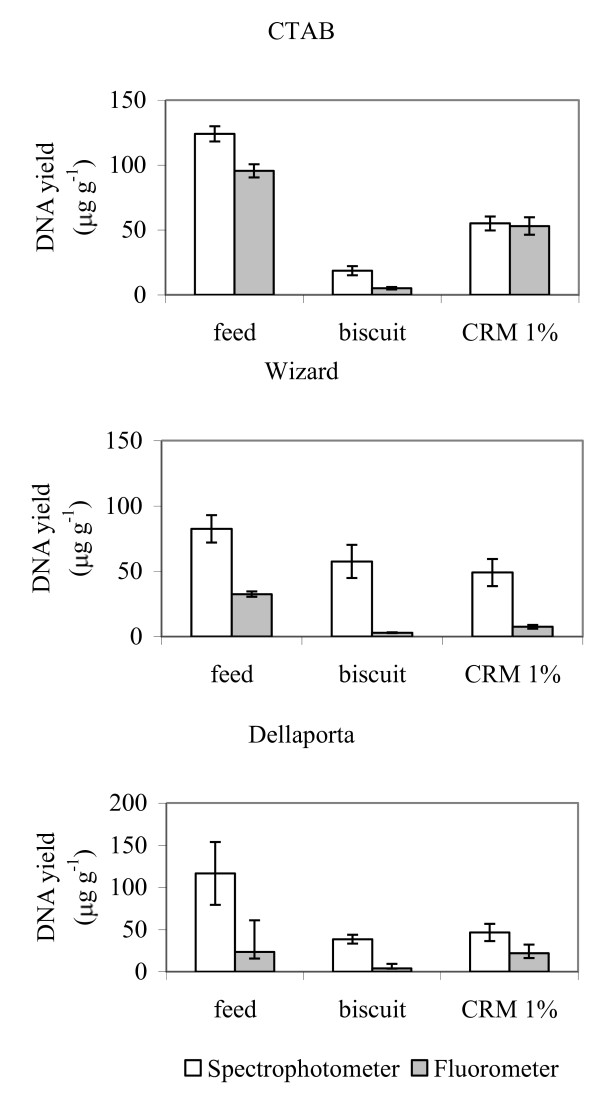
**Mean DNA yield (and standard error) determined for each combination of matrix (feed, biscuit or CRM 1%) and DNA extraction method (CTAB, Wizard or Dellaporta)**.

The mean range of variability in determination of the DNA yield by spectrophotometric and fluorometric measurements (see Figure 3) reflects the ability of the spectrophotometer to react with considerably higher amounts of solutes (from an average of 18.5 μg g^-1 ^in the CTAB/biscuit combination to an average of 116.3 μg g^-1 ^for Dellaporta/feed) than the fluorometer (from an average of 3.0 μg g^-1 ^for Wizard/biscuit to an average of 95.4 μg g^-1 ^for CTAB/feed).

Based on analysis-of-variance results (see Table 2), the estimated DNA yield is strongly affected by any of the factors (DNA extraction method, matrix and method used to estimate DNA concentration) at the levels employed in the experiment (*P *< 0.01).

**Figure 4:**
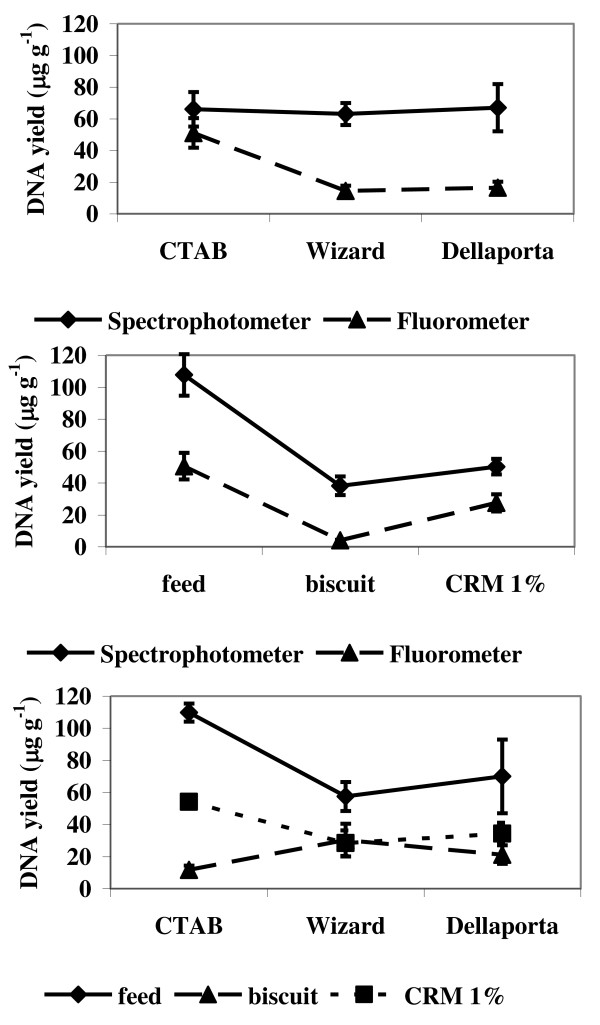
**Interaction effects (mean and standard error): DNA extraction method/method for DNA concentration (top), matrix/method for DNA concentration (middle), matrix/DNA extraction method (bottom)**.

The results in Table 2 show that two-way interactions are either highly significant (*P *< 0.01 for extraction/matrix and extraction/method for DNA concentration) or marginally significant (0.01 <*P *<0.05 for matrix/method for DNA concentration). This means that the simple effects are heterogeneous and any one factor cannot be assumed to be the same for each level of other factors. The graphs in Figure 4 investigate the dynamics of interactions between factors, with the non-parallel lines indicating interaction. The strong interaction between method to estimate DNA concentration and DNA extraction methods is quite clear in Figure 4 (top), with the fluorometer having a much more significant effect on the DNA yield from samples extracted by CTAB than by other extraction methods: DNA yield measured via UV absorbance (average of eighteen samples from the three matrices per extraction method) is rather stable across the three DNA extraction methods, hence the interaction shown by the couple CTAB/fluorometer could be explained either as a selective enrichment of dsDNA target in solution by the extraction method or as indication of interplay between Picogreen reagent and left-over compounds from CTAB procedure. Some synergic effect between feed and the spectrophotometer also emerges in Figure 4 (middle). Interactions between the matrix and the DNA extraction method are also clear in Figure 4 (bottom), where more DNA is yielded from feed and CRM 1% samples extracted by CTAB than from biscuit samples.

The three-way interaction is not significant (*P *> 0.2, see Table 2). Consequently, the simple interactions remain essentially the same in the presence of a third factor (e.g. matrix/method interaction under either a spectrophotometer or a fluorometer).

### RRS quantification

When the approach adopted by the EURL-GMFF for assessment of DNA quality was applied to the extracts, thus selecting for samples complying with the acceptance criteria for inhibition runs, the effect of alternative factors on GM concentration changed to some extent (see Table 3). In particular, the effect of the DNA extraction method turned out to be marginally significant (0.01 <*P *< 0.05). The effect exerted by the matrix remains significant, and the same is true for interaction effects (extraction/matrix, *P *< 0.01). As regards matrices, biscuit (2.1% on average after 'filtering') diverged from the others (1.2% for feed and 1.1% for CRM 1%).

**Figure 5:**
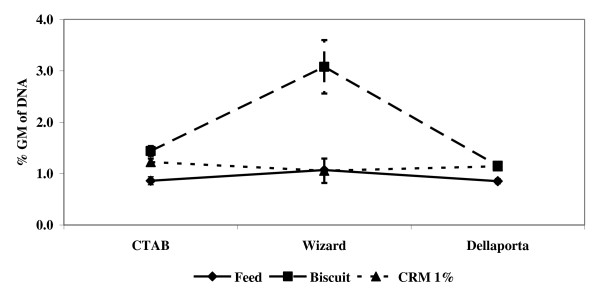
**Matrix/DNA extraction method interaction (mean and standard error); data violating the DNA quality acceptance inhibition tests were excluded**.

Further investigation of the interaction between extraction methods and matrices reveals no significant interaction patterns, except with the Wizard/biscuit combination (see Figure 5). The strong similarity between other combinations (1.1% of GM DNA as the overall mean) suggests that the uneven behaviour emerging from applying the Wizard extraction method to the biscuit matrix (mean value 3.1%) has the effect of distorting the statistical response. Importantly, further testing on extraction/matrix interactions carried out after excluding the Wizard/biscuit set of % GM concentrations (not shown in detail) found significant interaction between extraction methods and matrices only when all the data were processed (*P *< 0.01); in contrast when the quality of the DNA extracts was checked against the acceptance criteria (data not passing the inhibition run were removed) the interaction between the extraction method and the matrix became insignificant (*P *> 0.3) once again.

Finally, for each matrix and DNA extraction method, ISO validation metrics and PCR efficiency rates are reported in Table 4 (data violating the inhibition test criteria were excluded from the calculation) together with fuzzy logic-based aggregation measures. For matrices other than CRM 1%, bias values were calculated after estimation of the mean observed % GM following log transformation (0.87% for feed and 1.84% for biscuit). Amplification efficiencies are > 0.98 with each method, while it emerges that large bias and repeatability standard deviation values tend to be associated with the Wizard DNA extraction method (e.g. *B *> 80% with Wizard/biscuit). The other two methods comply with ENGL-based performance criteria (*B *< 25%, *RSD_r _*< 25%: http://http:/ / gmo-crl.jrc.ec.europa.eu/ doc/ Min_Perf_Requirements_Analytical_me thods.pdf). At the settings chosen for operation of the expert system, the poor performance of Wizard is particularly evident with a biscuit matrix, as distinctly reflected in the accuracy module (1.0000) and in the indicator (0.7500) of the fuzzy logic-based assessment method (see Table 4).

**Table 4:** Validation and real-time PCR measures computed with three different DNA extraction methods on three matrices

Validation/efficiency measures (and fuzzy-based modules and indicator)		DNA extraction method		
		
		CTAB	Wizard	Dellaporta
**CRM 1%**				

Basic measures	Average bias (*B*, %)	22.3	10.5	14.0
	Relative standard deviation repeatability (*RSD_r_*, %)	13.8	34.6	9.6
	Reference gene efficiency (*E_1_*, %)	99.4	99.8	99.8
	Target gene efficiency (*E_2_*, %)	99.7	99.5	99.2

Fuzzy-based aggregated measures	Accuracy	0.4271	0.5000	0.0000
	Efficiency	0.0000	0.0000	0.0000
	Indicator	0.2736	0.3750	0.0000

**Feed**				

Basic measures	Average bias (*B*, %)	0.8	23.5	1.6
	Relative standard deviation repeatability (*RSD_r_*, %)	20.5	-*	11.8
	Reference gene efficiency (*E_1_*, %)	99.4	99.8	99.8
	Target gene efficiency (*E_2_*, %)	99.0	98.5	99.2

Fuzzy-based aggregated measures	Accuracy	0.0011	-*	0.0000
	Efficiency	0.0000	0.0000	0.0000
	Indicator	~0.0000	-*	0.0000

**Biscuit**				

Basic measures	Average bias (*B*, %)	12.3	87.5	29.9
	Relative standard deviation repeatability (*RSD_r_*, %)	14.8	37.7	14.0
	Reference gene efficiency (*E_1_*, %)	99.7	99.9	99.8
	Target gene efficiency (*E_2_*, %)	99.7	99.8	99.2

Fuzzy-based aggregated measures	Accuracy	0.0000	1.0000	0.5000
	Efficiency	0.0000	0.0000	0.0000
	Indicator	0.0000	0.7500	0.3750

- Not including data violating DNA quality acceptance from inhibition tests.

- Estimated robust means are: feed, 0.87%; biscuit, 1.84%. Fuzzy logic is used to computed aggregated measures of *B *and *RSD_r _*(Accuracy), *E_1 _*and *E_2 _*(Efficiency), and an overall indicator

* Impossible to calculate repeatability and derived measures for lack of replicates.

## Discussion

The modular approach to method validation initially proposed by [9] and also discussed elsewhere [CO-EXTRA project, http://www.coextra.eu/research_themes/topics202.html, Codex Committee on methods of analysis and sampling, ftp://ftp.fao.org/codex/Alinorm10/al33_23e.pdf; 25], entails that each step of the analytical procedure is validated as a stand-alone module and needs to meet acceptance criteria before the output of one module is transferred as input to the next; its practical implementation would offer several advantages at enforcement level:

- laboratories could select the preferred and ideally validated DNA extraction procedure providing acceptable yield and DNA quality fit for the PCR-based module;

- DNA extraction protocols could be applied to different matrices provided the acceptance criteria set for DNA quality are met;

- the modular approach would make it possible to reduce the number of target-taxon reference systems in food analysis, particularly when multiple events from the same ingredient (i.e. same plant species) in a sample could be quantified in relation to only one validated taxon-specific system, with the practical consequence of favouring less laborious, but time-effective and cost-saving practices.

Incidentally, general endorsement of the modular approach would also support wider acceptance of flexible application of accreditations across regions and organisations, thus cutting several laboratory constraints and burdens [25].

Recently, the European Network of GMO Laboratories (ENGL, http://gmo-crl.jrc.ec.europa.eu/guidancedocs.htm) updated a guidance document on how methods for GMO analysis should be evaluated and validated in the context of the European legislation. It included a minimum set of acceptance criteria to appraise the quality of DNA extracts consisting mainly of running inhibition tests and applying stringent acceptance criteria. This is a move in the direction of underpinning the modularity principle and providing tools for critically combining the DNA extraction module with the analytical module (qPCR) in a single method. Generally speaking, criteria to appraise DNA fitness to access downstream qPCR applications should take in consideration three elements: *i*) DNA in reaction should be enough to result in efficient target amplification. This is achieved following the reaction set-up described in the validated method. If DNA yield is a limiting factor from the DNA extraction module, it is essential to load in reaction an amount of GM-target sequence above its limit of quantification (LOQ); *ii*) the DNA should be of sufficient length to be amplified in qPCR. This has basically to do with the amplicon length which are normally quite short in qPCR methodologies, therefore in case of highly processed matrices it is important to combine the DNA extraction method which does not add to the fragmentation state of DNA molecules; *iii*) finally, DNA extracts should be adequately free from PCR inhibitors. This is recognised as a relevant point by the scientific community and it is mentioned in ISO norms (20, 21).

A proposal to implement the modular principle and apply a quality 'filter' on DNA extracts, focusing on the reaction efficiencies of dilution series of DNA extracts, was also put forward independently [12].

Consequently, the primary aim of this study was to investigate whether different DNA extraction methods could be interchangeable and if DNA acceptance criteria could be applied to make the two modules independent.

The extraction of nucleic acids from biological material requires cell lysis, inactivation of cellular nucleases and separation of the desired nucleic acid from cellular debris. Three extraction methods were selected in this study due to their wide application in GMO field and plant molecular biology and to the difference on lyses buffer and on DNA isolation technique. The CTAB-based protocol (http://mbg.jrc.ec.europa.eu/capacitybuilding/docs-manual-EN.htm, for extensive review) is particularly suitable for the elimination of polysaccharides and polyphenolic compounds otherwise affecting the DNA purity. Plant cells can be lysed with the ionic detergent CTAB, which forms an insoluble complex with nucleic acids in a low-salt environment. Under these conditions, polysaccharides, phenolic compounds and other contaminants remain in the supernatant and can be washed away. The DNA complex is solubilised by raising the salt concentration and precipitated with ethanol or isopropanol; Dellaporta-derived method uses first a thermal lyses in the presence of Tris HCl, EDTA, CTAB and β- mercaptoethanol, followed by deprotenisation and removal of contaminants by phenol-chloroform precipitation; the Wizard™ ('Wizard') magnetic DNA purification for food kit makes use of a thermal lyses and magnetic bead technology for the DNA isolation in a mobile solid phase. Solid phase techniques simplify nucleic acid purification since they can replace several steps of centrifugation, organic extraction and phase separation with a single, rapid magnetic separation step.

The analysis performed on the full set of data - assessing three matrices and three extraction methods - revealed significant interaction effects that tended to decline (in probabilistic terms) when data reduction was performed by applying acceptance criteria to inhibition tests (see Table 3). However, such interaction effects (that is, violation of the assumption of module independence) were mainly due to application of the Wizard DNA extraction method to the biscuit matrix (see Figure 5) and disappeared when these data were excluded from the analysis, combined with application of quality criteria from inhibition tests. Wizard-based DNA extraction showed a significant overestimation of the % GM DNA concentration, particularly in the biscuit matrix (see Figure 5). Similar findings were reported by [14] on flours of certified reference material and by other authors [29] when the DNA extract was passed through the magnetic beads purification step. Alike results were obtained for a complex feedstuff, where the Wizard magnetic DNA extraction method introduced a bias in the ratio between the two analytes, with the results showing a significant increase (up to 20 times), much more than observed by [15] in flours of certified reference material with regard to the % GM obtained when the DNA extract was passed through the magnetic beads purification step (Berben, G., CRA-W, Gembloux, Belgium, personal communication).

This circumstance is also substantiated by the fuzzy logic-based assessment which revealed quite a high score (0.75) for the fuzzy-logic indicator (see Table 4), associated with lower performance from the biscuit/Wizard DNA extraction combination (i.e. high quantification bias).

Such analysis gives a clue to module independence. Analysis of variance found that applying data-filtering criteria before running the qPCR showed some effectiveness. It made it possible to assess the interaction between the factors involved in the process leading to GM DNA quantification. Although application of acceptance criteria to inhibition tests is certainly useful, its effectiveness for making the matrices independent of the DNA extraction method needs further investigation. Moreover, this can be achieved at the cost of excluding many data, e.g. those obtained when the Wizard extraction method was applied to feed (see Table 1). Cases like this are a clue that the wrong method is being applied to a given matrix.

In this study, low 260:230 ratios were observed (especially with Wizard but also with Dellaporta, when the latter was applied to feed and biscuit, see Table 1), possibly due to the presence of contaminants absorbing light at 230 nm (i.e. ethylenediaminetetraacetic acid, phenol and guanidine thiocyanate), but this was not related to the DNA yield. This study also considered whether the well-known laboratory practice to estimate the purity of DNA extracts based on absorbance ratios at defined wavelengths (260:280 and 260:230 in the ultraviolet spectrum) was in agreement with assessment of DNA quality by means of inhibition runs. Basically, no significant convergence was seen in this respect (see Table 1). Absorbance ratios of 260:280 and 260:230 have long been used in molecular biology to obtain an estimate of the purity of nucleic acid preparations intended for numerous applications, such as Southern and Northern blotting, reverse transcription, cloning and generation of libraries [30]. However, these common ways of assessing DNA purity are insufficient when it comes to decide whether a DNA extract is fit-for-purpose of application to qPCR, where parameters such as method linearity over a dynamic range and amplification efficiency come into play [[12,19] and http://gmo-crl.jrc.ec.europa.eu/doc/Min_Perf_Requirements_Analytical_methods.pdf]. The proposed acceptance criteria for assessment of DNA quality are hence intended to 'filter' for those DNA extracts suitable for downstream qPCR application.

Determination of the absorbance of a DNA solution in the UV spectrum - and conversion of the result into a concentration measurement - is a common technique. Recently fluorescence response of intercalating dyes, particularly the PicoGreen dsDNA quantitation reagent, are gaining more and more acceptance due to their reliability, sensitivity, extended dynamic range and lesser interaction with single-strand nucleic acids [31]. Consequently, this method for determining DNA concentration is becoming a well-established procedure in GMO testing laboratories too.

A recent study [32] found that UV and fluorimetric determination (average between the PicoGreen and the Hoescht dye methods of determining DNA concentration) agreed quite well when DNA concentration was measured from DNA samples extracted by the CTAB procedure from a relatively simple and unprocessed matrix such as NK603 certified reference material, at different percentages of GM content. However, once the CRM materials were processed by sonication or heat treatment this agreement was lost and, as the level of DNA degradation was forced, the amount of DNA measured increased when the UV method was used but, importantly, declined using the fluorescent dye method. The findings presented in this study mirror such behaviour and show that the combination of CTAB with 1% RRS certified reference material produced fairly close agreement between the estimates of DNA yields either via UV (260 nm) or PicoGreen (see Table 1). This study found different outcomes when other DNA extraction methods were used and DNA extracts were quantified by UV and fluorescence. Processed materials (biscuit and feed) showed the most striking deviations between spectrophotometric and fluorimetric results. This is probably due to the ability of short or single-strand nucleic acid fragments to interfere with UV rays more than with PicoGreen dye. A qualitative assessment of the DNA extracts by visual inspection of the results of the gel electrophoresis run (see Figure 2) does indeed agree more with the yield data from fluorescence methods than with those obtained from spectrophotometric determination. Gel analysis suggests that per each DNA isolation procedure, DNA extracted from the feed, and even more clearly from the biscuit, matrix suffered extensive damage compared to an unprocessed matrix (CRM 1%): the signs of broad degradation are quite striking, with DNA fragments dispersed everywhere between high and very low molecular weight. A tendency to select for low molecular weight DNA strands might be identified with the Wizard beads and Dellaporta methods. By contrast, DNA extracted with the CTAB and Dellaporta methods from certified reference material show a distinctly high molecular weight band.

Hence, the 'liberal' estimation of DNA content by UV could not adequately reflect the relative abundance of intact and amplifiable targets and could affect GMO quantification. In other words, if DNA is severely damaged the less abundant reaction target, typically the GM analyte, might fall close to or below the LOQ with practical consequences for the ability to quantify the target accurately. In order to quantify GMOs in food correctly, the degree of feed processing and the amount of degraded DNA or its average molecular weight are not paramount. Instead, the crucial factors are that the average length of the extracted DNA molecules must be longer than the amplicon sizes, the amount of the less abundant target sequence must be above the practical LOQ [33], there must be no imbalance between the GM/reference targets (if induced by industrial processing and/or extraction protocols) and there must be well-defined but affordable acceptance criteria for the quality of DNA extracts, as this study shows. Comparable reaction efficiencies between the food/feed sample and the reference curve [12] should also be considered further grounds for scientific consensus.

## Conclusions

The results of this study provide proof of concept for application of the modular approach to analytical methods in the field of GMO testing based on use of real-time PCR in food or feed samples. These had been chosen to represent an unprocessed material (CRM 1%), a raw mixed matrix (feed) and a processed mixed matrix (biscuit). Further investigation on additional processed materials would enlarge our understanding on the applicability of the modular approach proposed. By analogy, these could be extended to other fields of GMO testing. In particular, this study focused on the interactions between the DNA extraction method and real-time PCR analysis and found that DNA extracts should meet appropriate performance criteria if they are to be fit for analytical purposes, independent of the previous matrix/DNA extraction combination, with one significant limitation, namely when highly processed starting material (biscuit in this study) was combined with the magnetic beads system for DNA extraction used in this investigation.

To implement modular combination of the matrix/DNA extraction protocol and the analytical module, it is necessary to check the DNA quality by means of inhibition tests. The three performance parameters described in the section on 'Materials and methods' appear appropriate to assess whether the DNA extract is fit for purpose.

These investigations on the interactions between three matrices and three DNA extraction methods produced no evidence to support any relation between UV light absorbance ratios and inhibition run-based assessment of the quality of DNA extracts for target analyte quantification by RT-PCR technology. Certain association of poor DNA quality with either the highest or lowest DNA yields was fragmentarily observed but the evidence is insufficient to permit any general, statistically-sound conclusion on such a small number of cases.

## Authors' contributions

GB participated in the design of the study, drafted the manuscript, and performed the statistical analysis. CS interpreted the results and helped to draft the manuscript. MM, CS, RO and GVE conceived the study, participated in its design and coordination and in the interpretation of the data, and revised the manuscript. MDG, NF, EP and CD contributed towards acquisition of materials and carrying out of lab analyses (DNA extraction and purification, electrophoresis, spectrophotometric and fluorometric measures, real-time PCR). All authors read and approved the final manuscript.
